# Plasma soluble L-selectin in medicated patients with schizophrenia and healthy controls

**DOI:** 10.1371/journal.pone.0174073

**Published:** 2017-03-23

**Authors:** Satyajit Mohite, Fang Yang, Pooja A. Amin, Giovana Zunta-Soares, Gabriela D. Colpo, Laura Stertz, Ajaykumar N. Sharma, Gabriel R. Fries, Consuelo Walss-Bass, Jair C. Soares, Olaoluwa O. Okusaga

**Affiliations:** 1 UT Harris County Psychiatric Center, 2800 S MacGregor Way, Houston, Texas, United States of America; 2 Department of Psychiatry and Behavioral Sciences, University of Texas Health Science Center at Houston, Houston, Texas, United States of America; University of Texas Health Science Center at San Antonio Cancer Therapy and Research Center at Houston, UNITED STATES

## Abstract

Immune dysfunction has been implicated in the pathophysiology of schizophrenia. Leukocyte migration to the site of inflammation is a fundamental step of immune response which involves P-, E-, and L-selectins. Elevated selectin levels have been reported in un-medicated first-episode patients with schizophrenia but not in medicated patients with multi-episode schizophrenia. We measured fasting plasma soluble P-, E-, and L-selectin in 39 medicated patients with multi-episode schizophrenia and 19 healthy controls. In patients, psychotic symptom severity and cognitive function were assessed with the Positive and Negative Syndrome Scale (PANSS) and the NIH Toolbox Cognitive Test Battery respectively. C-reactive protein (CRP) and Body Mass Index (BMI) were measured in patients and controls. Comparison of selectin levels between patients and controls was done with t-tests and linear regression. Pearson correlation coefficients between plasma selectins and PANSS and cognitive measures were calculated. Geometric mean plasma soluble L-selectin level was lower in patients compared to controls from unadjusted (606.7 ± 1.2 ng/ml vs. 937.7 ± 1.15 ng/ml, p < 0.001) and adjusted analyses (β = 0.59; CI 0.41 to 0.88, p = 0.011). There was a trend towards higher plasma soluble P-selectin in patients compared to controls (90.4 ± 1.2ng/ml vs. 71.8 ± 1.2ng/ml, p = 0.059) in the unadjusted analysis. There was no association between the selectins and psychotic symptoms or cognitive function in the patients. In addition, the selectins were not significantly associated with CRP or BMI. The limitations of this study include small sample size and unavailability of information on medications and blood cell counts. The potential utility of soluble L-selectin as a biomarker of antipsychotic exposure in patients with schizophrenia and the concomitant change in immune response with the use of antipsychotics should be further evaluated.

## Introduction

Findings from previous studies suggest that peripheral inflammation may lead to the activation of immune responses in the central nervous system [1,2], which may involve the induction of cytokines and adhesion molecules, recruitment of immune cells, and the activation of microglia [3,4]. Schizophrenia, a severe mental illness characterized by delusions, hallucinations and thought disorder, is associated with elevated levels of peripheral and central markers of inflammation including raised plasma and CSF cytokines and microglial activation [5,6]. However, the link between peripheral and central inflammation is not well understood in schizophrenia. Endothelial immune activation has potential relevance in the transmission of inflammation from the periphery to the brain but has not been adequately studied in schizophrenia. Endothelial immune function includes the expression of heparan sulfate and selectins which are involved in the adhesion of leukocytes to inflamed endothelium [7–9]. Findings from animal studies have shown that cell adhesion molecules of the selectin family are essential in the spread of inflammation from the periphery to the brain in the context of liver inflammation [10,11].

Selectins are glycoprotein adhesion molecules located on leukocytes (L-selectins), platelets (P-selectins) and endothelial cells (P and E-selectins) [12]. The proteolyzed form of selectins in the circulation are called soluble selectins (sL/sP/sE selectins) [13]. Within the inflammation cascade, selectins are actively involved in the leukocyte-endothelial interaction, neutrophil recruitment, signaling, capture and rolling [14]. Increased serum sL-selectin levels have been found in various medical conditions including HIV infection [15], sepsis [16], atopic dermatitis [17], diabetic retinopathy [18], and bladder cancer [19]. Aberrant levels of sP-selectin have also been found in in autism and acute psychosis [20,21].

To our knowledge the only 2 studies [21,22] that have evaluated blood selectin levels in patients with schizophrenia have involved patients with first-episode psychosis who were not being treated with antipsychotic medications at the time of blood draw. However, based on the results of previous studies that have indicated that antipsychotics may influence immune response [23,24], we have hypothesized that unlike the findings in unmedicated first-episode patients, plasma selectins in medicated patients with multi-episode schizophrenia will be reduced in comparison with levels in healthy controls. This study is a comparison of plasma soluble L-, P-, and E-selectins in medicated patients with multi-episode schizophrenia and healthy controls. We also aimed to evaluate the association of plasma soluble selectins with psychotic symptoms and cognitive function in the patients.

## Materials and methods

### Patient sample

This study was carried out in accordance with the latest version of the Declaration of Helsinki. The institutional review board of The University of Texas Health Science Center, Houston, Texas, approved this study. A cross sectional design was used to compare plasma selectin levels of patients with schizophrenia and healthy controls. Plasma C-reactive protein was also measured in patients and controls as a general marker of inflammation in both groups. Body mass index (BMI), an important modulator of inflammation was also evaluated in patients and controls.

The patient sample was recruited from an inpatient setting affiliated with a University Department of Psychiatry. None of the patients were having first episode psychosis. All the patients had experienced multiple episodes of psychotic exacerbation with more than one hospital admission and were receiving antipsychotic treatment at the time of recruitment. Healthy controls were recruited from the nearby communities. Demographic data gathered included age, race, gender and level of education. Our inclusion criteria for patients were: a) DSM-5 diagnosis of schizophrenia; b) age 18 to 60 years; c) negative pregnancy test in females. The exclusion criteria were a) pervasive developmental disorder, dementia, delirium, other cognitive disorders; 2) current suicidal and homicidal ideations; 3) Urine drug screen positive for psycho-stimulants such as cocaine, amphetamines and ecstasy; 4) any infection, neoplasm, autoimmune disease or other primary inflammatory condition; 5) current use of any non-steroidal anti-inflammatory drugs; 6) current or anticipated corticosteroid use; 7) recent use of warfarin or any anticoagulant.

The study was described to all the participants after which written informed consents were obtained. The diagnosis of schizophrenia in the patient group was confirmed with the Mini-International Neuropsychiatric Interview (MINI).

### Blood sample

Overnight fasting venous blood was drawn from patients and controls in EDTA-containing tubes, and plasma was isolated and stored at -80°C until analyzed for selectins.

### Plasma selectin measurement

Enzyme-linked immunosorbent assay (ELISA) kits (RayBiotech, Inc. Georgia, USA) were used to analyze plasma levels of selectins in patients and controls, according to the manufacturer’s instructions. Samples were diluted 1:50 for the measurement of P- and E-selectins and 1:500 for L-selectin. Optical density values for the samples were then compared to standard curves ranging from 0–30ng/ml (P-selectin), 0–18pg/ml (E-selectin), and 0–25 ng/ml (L-selectin). Sensitivities of the assays were 20 pg/ml, 30 pg/ml, and 100 pg/ml for P-, E-, and L-selectin, respectively.

Elisa kit (Sigma-Aldrich, Missouri, USA) was used to analyze plasma levels of CRP in patients and controls according to the manufacturer’s instructions. Samples were diluted 1:20,000. Optical density values for the samples were then compared to standard curves ranging from 0–600pg/ml. The minimum detectable dose of Human CRP in this assay is 2pg/ml.

### Psychotic symptom and cognitive assessment in patients

Psychotic symptom severity was assessed with the Positive and Negative Syndrome Scale (PANSS) [25]. Cognitive function was evaluated with the National Institute of Health -NIH Toolbox Cognitive Test Battery [26]. The NIH Toolbox Cognitive Test Battery consists of the following cognitive measures: a) Dimensional Change Card Sort and Flanker Inhibitory Control and Attention Tests which measure executive function; b) Picture Sequence Memory Test which measure episodic memory; c) Picture Vocabulary Test and Oral Reading Recognition Test which measure language skills: d) Pattern Comparison Processing Speed Test which measures processing speed: e) List Sorting Working Memory Test which measures working memory. Fully adjusted scores on the NIH Toolbox Cognitive Test Battery were collected for each patient. Fully adjusted score compares the score of the test-taker to those in the NIH Toolbox nationally representative normative sample, while adjusting for key demographic variables (age, gender, race, ethnicity, and educational attainment) collected during the Toolbox national norming study [26].

### Statistical analyses

Data analysis was done using IBM SPSS version 20 (IBM CorP, Armonk, NY). Plasma selectin values were skewed to the right, and we therefore carried out logarithmic transformation (base 10) of individual values in an attempt to normalize the data. By calculating the anti-log of log-transformed mean values, geometric means with 95% confidence interval were obtained. T-test and chi-square tests were used to compare demographic and clinical (CRP and BMI) variables between patients and healthy controls. Unadjusted comparisons of plasma soluble selectin levels between patient and control groups were done with t-tests. Linear regression was used for the adjusted comparison between the two groups. We evaluated the association between plasma soluble selectin levels and psychotic symptoms and cognitive function respectively by calculating Pearson correlation coefficients between the selectins and PANSS and NIH Toolbox scores. We also evaluated the correlation between CRP and the selectins. All significance levels reported are two-sided and to reduce the chances of obtaining false-positive results (type I errors) we applied the Bonferroni correction [27] for the correlational analyses.

## Results

The comparison of demographic variables in patients with schizophrenia (n = 39) and healthy controls (n = 19) is shown in Table 1. Both groups were significantly different in gender distribution and education level. There were more males in the patient group (74.4%) than the control group (31.6%, p = 0.004). Educational attainment was lower in the patient group and more participants (88.6%) had educational attainment below the college graduation level in comparison to only 1% in the control group (p<0.001). Though not statistically significant, the patient group had a slightly lower mean age (32.79 ± 12.05 years), with more participants of black race (48.7%), in comparison to the control group, which had a mean age of 33.68 ± 9.9 years and less participants of black race (26.3%) %. BMI and CRP did not differ between patients and controls.

**Table 1:** Comparison of demographic characteristics of patients and control group.

	Patients with schizophrenia	Healthy Controls	p value
n (%)	39 (67.2%)	19 (32.8%)	
Mean Age ± SD	32.79 ± 12.052	33.68 ± 9.900	0.781
Gender			**0.004**
Male n (%)	29 (74.4%)	6 (31.6%)	
Female n (%)	10 (25.6%)	13 (68.4%)	
Race			0.456
White n (%)	11 (28.2%	7 (36.8%)	
Black n (%)	19 (48.7%)	5 (26.3%)	
Hispanic n (%)	8 (20.5%)	6 (31.6%)	
Asian n (%)	1 (2.6%)	1 (5.3%)	
Education Level*			**<0.001**
No high-school graduation n (%)	7 (20.0%)	0 (.0%)	
Graduated high-school (or equivalent) n (%)	10 (28.6%)	0 (.0%)	
Part college n (%)	14 (40.0%)	1 (5.3%)	
Graduated 2 years college n (%)	2 (5.7%)	8 (42.1%)	
Graduated 4 years college n (%)	2 (5.7%)	8 (42.1%)	
Part graduate/professional school n (%)	0 (.0%)	2 (10.5%)	
Mean BMI ± SD	27.27 ± 5.19	28.70± 5.05	0.359
Geometric mean CRP	0.19 ± 0.35	0.16 ± 0.30	0.678

Footnotes: SD = standard deviation, BMI = Body mass index, CRP = C-reactive protein.

* 4 patients had missing data on education level.

### Plasma selectin levels in patients and controls

**Fig 1:**
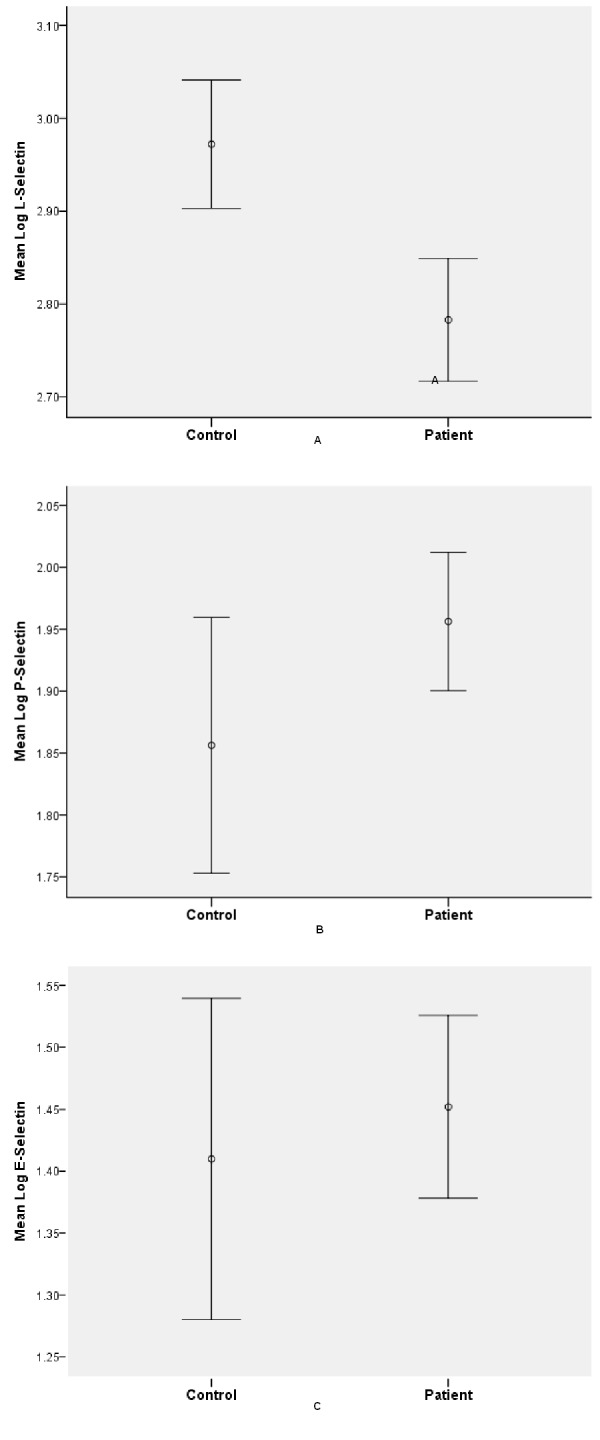
**Plasma soluble L-selectin (A), P-selectin (B) and E-selectin (C) levels in patients with schizophrenia and healthy controls.** Error bars are representing 95% confidence intervals, and the little circle stands for the mean of the log-transformed value of each selectin. Plasma soluble P-, E- and L-selectin concentration were log transformed to normalize the data. Geometric means reported in the text were calculated by exponentiating mean log-transformed P-, E- and L-selectin for adjusted and unadjusted comparisons of patients and controls.

From unadjusted analyses, plasma sL-selectin was lower in patients than in controls (606.7 ± 1.2 ng/ml vs. 937.7 ± 1.15 ng/ml, p < 0.001), a finding that persisted after adjusting for age, gender, race and education (β = 0.59; CI 0.41 to 0.88, p = 0.011). There was a trend towards higher plasma sP-selectin in patients compared to controls (90.4 ± 1.2ng/ml vs. 71.8 ± 1.2ng/ml, p = 0.059) in the unadjusted analyses but the adjusted analyses indicated that sP-selectin and sE-selectin did not differ between patients and controls (Fig 1A, 1B and 1C and Table 2).

**Table 2:** Comparison of selectin levels in patients and control group.

Geometric mean ± SD (ng/ml)	Patients with schizophrenia (n = 39)	Healthy Controls (n = 19)	P value
sL-selectin	606.7 ± 1.2	937.7 ± 1.2	<0.001
sP-selectin	90.4± 1.2	71.8 ± 1.2	0.059
sE-selectin	28.3 ± 1.2	25.7 ± 1.2	0.531

Footnotes: SD = standard deviation.

### Association of selectins with psychotic symptoms and cognitive function

There was no correlation between any of the selectins and PANSS sub scores (positive, negative and general psychopathology sub scores) or total scores (S1 Table). Also, none of the selectins correlated with any of the NIH Toolbox cognition measures (Pearson correlations have been provided in S2 Table).

### Correlation of selectins with CRP and BMI

There was no correlation between any of the selectins and CRP or BMI in patients and controls (Correlations provided in S3 Table).

### Comparison of selectins by gender in patients and controls

In patients, selectin levels did not differ between males and females but in the healthy control group, soluble P-selectin levels were higher in males vs. females (S4 Table).

## Discussion

The main result of this study is that mean plasma sL-selectin level in medicated patients with schizophrenia is lower in comparison to the mean level in healthy controls. In the comparison of selectin concentration between males and females in the patient and control groups respectively, we found that sP-selectin was higher in male controls than female controls. There was no significant difference in sP and sE- selectin levels between patient and control groups. Iwata et al. did a similar study in unmedicated patients with schizophrenia and found increased sL-selectin levels in the patients [22]. In contrast, all the patients enrolled in our study were receiving various antipsychotic medications at the time they were enrolled into this study. The possible impact of antipsychotic medication on immune function and subsequent alterations of inflammatory markers (e.g. Interleukins) has been suggested earlier [28–29]. Such alterations were seen acutely after 9 days, and also late after 8 weeks of antipsychotic treatment [28,30]. In comparison with the sL-selectin levels in the Iwata et al. study and this study, the levels seem similar in healthy controls but are lower in our sample of medicated patients. Therefore, effects of antipsychotic medication on immune function and inflammatory response might have contributed to the different results between ours and the previous study of Iwata et al. [22]. This line of reasoning has to be further evaluated in future studies.

Considering the fact that the selectins are involved in the initial contact of circulating immune cells with the vascular endothelium in a multi-step process which can eventually lead to the transfer of immune cells across the blood brain barrier into the CNS [31], the finding of reduced sL-selectin in medicated patients maybe a significant outcome related to the pathophysiology of the illness. Patients with schizophrenia as well as individuals at ultra-high risk for psychosis have been found to have elevated microglial activity (an indicator of CNS neuroinflammation) [6,32,33]. Though speculative, it may be that high levels of soluble L-selectin seen in unmedicated patients fosters the spread of immune cells from the periphery to the brain (leading to activation of the resident microglial cells) and antipsychotic medications may counteract this effect by lowering the levels of soluble L-selectin and potentially reducing CNS neuroinflammation. This hypothesis should also be tested in futures longitudinal studies.

In the current sample of patients with schizophrenia, none of the selectins were related to the severity of psychotic symptoms or to cognitive function. Iwata et al also did not find any association between symptom severity and serum soluble L-selectin in their sample of unmedicated patients with schizophrenia. To our knowledge, no previous study has evaluated the association of selectins with cognitive function assessed with the NIH toolbox cognitive test battery.

There was a statistical trend (p = 0.059) towards higher sP-selectin in our sample of patients in comparison with healthy controls. A possible explanation is that psychosis promotes a pro-coagulable state since sP-selectin reflects activation of platelets and endothelium; the explanation we have proffered is purely speculative since we do not have data on platelets or endothelium. sP-selectin levels in our sample of patients and controls are lower than the patients and controls in the study by Masopust et al (90.4 and 71.8 vs. 209.9 and 112.4 respectively). A possible reason for this difference in values is that we have reported geometric mean values whereas Masopust et al. reported arithmetic mean values.

Strengths of this study include the use of a validated structured interview to confirm the diagnosis of schizophrenia in cases and adjustments were made for confounders including age, sex, race and education. Inability to control for the type, dosage and duration of antipsychotic treatment as well as lack of information on duration of illness are limiting factors in this study. Another limitation of this study is its cross sectional design which makes it impossible to evaluate the potential utility of the selectins as trait versus state markers in schizophrenia. Small sample size is another limitation of this study. Although we have included data on CRP and BMI, other factors that could potentially modulate inflammation such as smoking, exercise, and blood cell counts were not controlled for.

## Conclusion

Given the key role of selectins in the inflammation cascade, reduced plasma soluble L-selectin levels in medicated patients with schizophrenia may be indicative of a concomitant change in immune response. The findings of this study suggest a modifying effect of antipsychotic medications on L-selectin and if replicated in future studies, plasma soluble L-selectin may become a biomarker of antipsychotic exposure in patients with schizophrenia. Other confounders including duration of the illness, smoking, exercise and blood cell counts need to be addressed in future studies.

## Supporting information

S1 Table Correlation between the individual selectins and psychotic symptoms. (DOCX)

S2 Table Correlation between the individual selectins and NIH Toolbox Cognitive Measures. (DOCX)

S3 Table Correlation between the individual selectins and CRP and BMI. (DOCX)

S4 Table Comparison of plasma selectins between male and female patients and controls respectively. (DOCX)

S1 Data Data file. (XLSX)
