# Supplementary material for: Plasma soluble L-selectin in medicated patients with schizophrenia and healthy controls
Source: PLoS One. 2017 Mar 23;12(3):e0174073. doi: 10.1371/journal.pone.0174073 (PMC5363914; doi:10.1371/journal.pone.0174073)
Supplement: S1 Table — (DOCX) [file pone.0174073.s001.docx]

**Supporting Information:**

**S1 Table: Correlation between the individual selectins and psychotic symptoms**

| **Selectins** |  | P-positive | P-negative | P-general | P-total |
| --- | --- | --- | --- | --- | --- |
| P-selectin | Pearson Correlation | -0.18 | 0.13 | -0.05 | 0.10 |
|  | Sig. (2-tailed) | 0.35 | 0.48 | 0.78 | 0.59 |
| E-selectin | Pearson Correlation | -0.20 | -0.05 | 0.00 | 0.03 |
|  | Sig. (2-tailed) | 0.30 | 0.80 | 0.00 | 0.88 |
| L-selectin | Pearson Correlation | -0.06 | -0.17 | -0.15 | -0.18 |
|  | Sig. (2-tailed) | 0.75 | 0.36 | 0.41 | 0.34 |

Footnotes: PANSS= positive and negative syndrome scale, P-positive= PANSS Positive subscale, P-negative= PANSS Negative subscale, P-general= PANSS general subscale scores, P-total= PANSS Total score.
