# Supplementary material for: Plasma soluble L-selectin in medicated patients with schizophrenia and healthy controls
Source: PLoS One. 2017 Mar 23;12(3):e0174073. doi: 10.1371/journal.pone.0174073 (PMC5363914; doi:10.1371/journal.pone.0174073)
Supplement: S2 Table — (DOCX) [file pone.0174073.s002.docx]

.

**S2 Table: Correlation between the individual selectins and NIH Toolbox Cognitive Measures**

| **Selectin** |  | **CC** | **TC** | **FC** | **PV** | **FL** | **LS** | **DC** | **PC** | **PS** | **OR** |
| --- | --- | --- | --- | --- | --- | --- | --- | --- | --- | --- | --- |
| **P-selectin** | Pearson Correlation | -0.36 | -0.24 | 0.07 | -0.13 | 0.39 | -0.02 | -0.00 | 0.11 | -0.25 | 0.00 |
|  | p-value | 0.11 | 0.29 | 0.74 | 0.53 | 0.05 | 0.91 | 0.99 | 0.59 | 0.24 | 0.99 |
| **E-selectin** | Pearson Correlation | -0.31 | -0.11 | 0.24 | 0.02 | 0.44 | 0.15 | 0.22 | 0.19 | 0.03 | 0.15 |
|  | p-value | 0.18 | 0.65 | 0.25 | 0.91 | 0.03 | 0.50 | 0.29 | 0.39 | 0.88 | 0.48 |
| **L-selectin** | Pearson Correlation | 0.01 | 0.22 | 0.27 | 0.38 | 0.04 | -0.01 | 0.30 | 0.32 | 0.28 | -0.14 |
|  | p-value | 0.98 | 0.33 | 0.20 | 0.06 | 0.84 | 0.96 | 0.15 | 0.13 | 0.17 | 0.52 |

CC = Cognition Crystallized Composite**,** TC= Cognition Total Composite Score**,** FC= Cognition Fluid Composite**,** PV= Picture Vocabulary**,** FL= Flanker Inhibitory Control and Attention**,** LS= List Sorting Working Memory**,** DC= Dimensional Change Card Sort**,** PC= Pattern Comparison Process Speed**,** PS= Picture Sequence Memory**,** OR= Oral Reading Recognition
