# Supplementary material for: Plasma soluble L-selectin in medicated patients with schizophrenia and healthy controls
Source: PLoS One. 2017 Mar 23;12(3):e0174073. doi: 10.1371/journal.pone.0174073 (PMC5363914; doi:10.1371/journal.pone.0174073)
Supplement: S3 Table — (DOCX) [file pone.0174073.s003.docx]

**S3 Table: Correlation between the individual selectins and CRP and BMI**

|  |  | P-selectin | E-selectin | L-selectin |
| --- | --- | --- | --- | --- |
| CRP | Pearson Correlation | 0.08 | -0.13 | -0.22 |
|  | p-value | 0.57 | 0.39 | 0.12 |
| BMI | Pearson Correlation | -0.09 | 0.09 | 0.03 |
|  | p-value | 0.56 | 0.57 | 0.81 |

CRP = C-reactive protein, BMI= Body mass index
