# Supplementary material for: Plasma soluble L-selectin in medicated patients with schizophrenia and healthy controls
Source: PLoS One. 2017 Mar 23;12(3):e0174073. doi: 10.1371/journal.pone.0174073 (PMC5363914; doi:10.1371/journal.pone.0174073)
Supplement: S4 Table — (DOCX) [file pone.0174073.s004.docx]

**S4 Table: Comparison of plasma selectins between male and female patients and controls respectively**

| **Selectins** | **Group** | **Gender** | **Mean** | **S.D.** | **p value** |
| --- | --- | --- | --- | --- | --- |
| P selectin | Cases | Male | 89.53 | 1.49 | 0.76758 |
|  |  | Female | 93.81 | 1.20 |  |
|  | Control | Male | 98.91 | 1.45 | **0.033** |
|  |  | Female | 61.21 | 1.58 |  |
| E selectin | Cases | Male | 26.50 | 1.63 | 0.69041 |
|  |  | Female | 36.17 | 1.46 |  |
|  | Control | Male | 28.30 | 1.63 | 0.610 |
|  |  | Female | 24.48 | 1.94 |  |
| L selectin | Cases | Male | 597.14 | 1.49 | 0.64353 |
|  |  | Female | 643.29 | 1.75 |  |
|  | Control | Male | 1042.24 | 1.67 | 0.33684 |
|  |  | Female | 889.38 | 1.19 |  |

Footnotes: SD= standard deviation
